# Patterns and predictors of malaria among head porters: a mobile population in Ghana

**DOI:** 10.1186/s12936-024-05000-2

**Published:** 2024-07-27

**Authors:** Eunice Mintah-Agyeman, Boakye-Yiadom Adomako, George Asumah Adu, Benjamin Oteng, Fergusson Duvor, Ihsan Abubakari, Mildred Kommey, Alexander Asamoah, Nana Yaw Peprah, Keziah L. Malm

**Affiliations:** National Malaria Elimination Programme, Public Health Division, Ghana Health Service, Accra, Ghana

**Keywords:** Head porter, Malaria infection, *Plasmodium falciparum*, Predictors, Health-seeking behaviour

## Abstract

**Background:**

Head porters popularly known as ‘*Kayayei*s in Ghana, face challenges in accessing essential health care services due to the mobile nature of their trade, low formal education, poor settlements, low-income among others. Kayayeis are predominantly females and form part of the mobile population who are at increased risk of malaria infection. Despite their increased risk of malaria, mobile populations are difficult to target for malaria interventions, hence serving as potential drivers of transmission even if malaria in the general population is controlled. The study, therefore, assessed the patterns and predictors of malaria among the Kayayei population in Ghana to inform policy decisions.

**Methods:**

A mixed methods study was conducted among Head-porters and their leaders in the three main hubs of Head-porters in Ghana; namely Accra, Kumasi, and Tamale. Blood samples were collected from participants and tested for malaria parasites using Rapid Diagnostic Test (RDT). Additionally, data including socio-demographics, malaria knowledge, attitude and practice were collected using a semi-structured questionnaire. Associations between malaria status and participants characteristics were determined by logistic regression (p < 0.05). Thematic analysis was used to analyse transcripts from the key informant interviews.

**Results:**

Out of 754 head porters studied, 10.48% (79) tested positive for malaria. The majority 43.10% (325/754) of the head porters were twenty years and below, and most 67.11% (506/754) had no formal education. Nearly half (50.4%) were not on any health insurance. Receiving malaria education in the past 6 months [AOR = 0.48, (0.26–0.88), p-value 0.02], and having poor knowledge of malaria [AOR = 2.23, (1.26–4.27), p < 0.02], were the factors significantly associated with malaria infection.

**Conclusion:**

The prevalence of malaria among ‘Kayayei’s was estimated at 10.46%. A majority of them sleeps outside and in structures without mosquito screens. Receiving malaria education in the past 6 months reduced the odds of malaria infection whilst poor knowledge of malaria increased the odds of malaria infection among the porters. The authors recommend that the National Malaria Elimination Programme and partners should provide long-lasting insecticidal nets (LLIN) and other outdoor interventions for use by this special group. Designated state institutions should arrange free National Health Insurance Scheme (NHIS) registration for ‘Kayayeis’ to narrow the health access gap.

## Background

Malaria remains a global public health concern despite the plethora of interventions towards its elimination [1]. There were an estimated 247 million cases of malaria from 84 malaria endemic countries most of which are in the WHO African region. This was an increment from the estimated 245 million cases of malaria in 2020 [1]. Ghana ranks among the top 15 nations globally in terms of malaria burden, accounting for 2.2% of global malaria cases as well as 2% of global malaria-related fatalities. It constitutes around 4% of malaria cases in the West African region [2]. Notwithstanding, Ghana has demonstrated notable advancements in the field of malaria control in the past decade (2011–2022). Malaria prevalence reduced from 27.5% in 2011 [3] to 8.6% in 2022 [4] and confirmed malaria cases per 1000 population reduced from 192 in 2019 to 159 per 1000 in 2022. Consequently, malaria deaths also reduced from 2,799 deaths in 2012 to 151 deaths in 2022. Despite this remarkable progress, malaria continues to have a devastating impact on people’s health and livelihoods, with varied transmission patterns across persons, seasons, and geographical areas.

Global migration statistics for the past 30 years show a rise in the proportion of female migrants [5]. This trend is nearly the same across sub-Saharan Africa, and a major characteristic is that migrants migrate to urban areas [5]. Human population movement (HPM) has been described as a major challenge to malaria elimination efforts, as it requires understanding of how the spatial distribution of malaria shifts through time and across multiple locations that become interconnected through population movements [6]. Mobile populations from high transmission zones risk reintroduction and reappearance in malaria-free receptive areas and has compromised eradication efforts in the past [7]. In endemic settings, understanding the patterns of parasite movements from local hotspots of transmission can provide valuable information for control by identifying both the regions where imported infections originate and where they may contribute substantially to transmission [8]. Reasons accounting for the rise in mobility are rural–urban migration in search of jobs and greener pastures, conflicts, and natural disasters such as floods [9]. In low-middle-income countries, migrants are confronted with health needs which affect the promotion of their well-being and healthy lives [10]. However, not much is known about the health needs of migrant female head porters (Kayayei) in Ghana. Head porters, popularly known as ‘Kayayeis’ in Ghana, are part of the mobile populations at risk of malaria infection. Head porters are mostly migrants from villages in the northern part of Ghana often without proper accommodation in the cities where they ply their trade. Despite their increased risk of malaria infection, head porters and other mobile populations are difficult to target for malaria interventions, hence serving as potential drivers of transmission even if malaria in the general population is controlled. The National Malaria Elimination Programme (NMEP) conducted this study to determine malaria prevalence and associated factors among head porters in Ghana to inform decision-making.

## Methods

### Study design

This study utilized a mixed methods approach of both qualitative and quantitative methods where the quantitative method was used to assess the prevalence of malaria and associated factors among ‘Kayayeis’ and the qualitative aspect was used to obtain information from their leaders regarding some social amenities, malaria burden, and prevention as well as health-seeking behaviour of ‘Kayayei’s’.

### Study area

This study focused on head porters and their leaders in the three (3) main hubs of head porters in Ghana namely: Northern zone (Tamale), Middle zone (Kumasi) and Southern zone (Accra). In each city a number of markets were randomly selected for the study as follows; Tema Station/Makola and Malata in Accra, Aboabo, Bombay and KTI in Kumasi, and Central Market in Tamale.

#### Tamale

Tamale is the capital city of the Northern Region of Ghana. It is the fourth-largest city in Ghana and has a population of 374,744 [9]. Due to its central location, Tamale serves as a hub for all administrative and commercial activities in the Northern region and the entire northern zone of Ghana. Tamale has seen significant growth and economic transformation in recent times; and this is evident by the rush of various companies to open branches in the city. This rapid economic growth has rendered it very favorable for head porting activities. There are two major markets in Tamale where these porters operate their trade: the Tamale Central Market and Aboabo Market. The Tamale Central Market which was randomly selected for this study is located at the heart of Tamale. Built in the nineteenth century, it is the oldest market in Tamale. Aside groceries and livestock, which are the main trading items in the market, there is also a big transport station located in the market, from where various buses and cars transport traders and their wares, as well as passengers to different parts of the country. This dual role played by the market does not only create human and vehicular congestion, but also makes it a lucrative area for head porting in the city.

#### Kumasi

Kumasi is in the middle zone of Ghana. It is the capital city of the Ashanti Region, with a population of 443,981, and the second largest city in Ghana [9]. It is alternatively known as "The Garden City" because of the many beautiful species of flowers and plants it had some years ago. Kumasi is approximately 500 kms north of the Equator and 200 kms north of the Gulf of Guinea. The Central Business District of Kumasi including areas like Adum, Bantama and Bompata (popularly called Roman Hill) is concentrated with lots of economic activities including banking, pottery, clothing and textile manufacturing making it a favourable location for head porting (Kayaye) activities. Aboabo, Bombay and KTI were the three markets randomly selected for this study. These markets are submarkets within the Kumasi Central Market, popularly known as Kajetia Market, which is the biggest open-air market in Kumasi. It has over 10,000 stores and stalls and rated as the largest single market in West Africa. This is an important economic hub, seeing traders in their thousands on daily basis from all corners of Ghana, West Africa and beyond. The constant movement of goods to and for market makes it suitable area for the head porting business.

#### Accra

Accra is the capital city of both the Republic of Ghana and the Greater Accra Region (GAR). It has an estimated population of 284,124 [9]. Accra serve as the economic and administrative hub of the Greater Accra Region. The central business district of Accra contains the city's main banks and department stores, as well as an area known as the Ministries, where Ghana's government administration is concentrated. Accra is endowed with both day and night economic activities, such as banking, trading, fishing, processing of wood into lumber or plywood, food production and manufacturing of other several items such as textiles, foam products, soaps and detergents, medicines, chemicals, rubber products, building materials. These economic activities make head porter activities a lucrative venture in Accra.

Tema Station/Makola and Malata markets were selected for this study from this zone. These constitute the main wholesale and retail market, in the principal commercial centre of the country. These markets trades in array of goods, ranging foodstuffs, clothing and accessories, cosmetics, car parts among others. There is significant presence of head porters owing to their scale and location.

### Sample size estimation

A respondent-driven sampling (RDS) technique was employed for this study. Using the respondent-driven sampling (RDS) technique for mobile population with recommended minimum design effect of 2 [11]. Using the formula, n = DE * [(z2pq)/d2] with n = sample size; p = estimated proportion of malaria among female head porters according to [12] was 33% (0.33); q = 1−p; z = 1.96 and d = 5% at 95% confidence interval and a design effect (DE) of 2, a minimum sample size used for this study was estimated at 680. Accounting for 10% non-response rate, the total sample size was 748 participants.

### Sampling procedure

Multi-staged and purposive sampling were employed for this study. Multi-stage was used to select the porters for the quantitative arm, whilst purposive sampling was used for selecting their leaders for the key informant interviews. In selection of the porters, an estimated number of head porters in each of the three zones was first obtained from heads of the porters and used to apportion proportion of porters to be sampled from in each city. Subsequently, based on the number of porters to be sampled per city and the population of porters in markets located in the city, we selected two markets in Accra, three in Kumasi and one in Tamale for the study. On the porter’s selection, an announcement was made by their leaders ahead of the survey for convergence at point(s) in the market. Any porter who was available and consent to participate in the study was enrolled until sample size was met. For the qualitative aspect, Key Informant Interviews (KIIs) was granted leader of the head porters in each of the six selected markets.

### Data collection and laboratory technique

#### Quantitative phase

A semi-structured questionnaire deployed on the ODK application was used to collect data from the porters at a centralized location in the market. Data including socio-demographics, knowledge, attitude and practices on malaria, presence of fever and malaria history. After the questionnaire was administered, finger prick blood samples were taken from the participants to test for the presence or absence of malaria parasites using RDTs (Pf/HRP2). Participants who tested positive for malaria were given first-line anti-malarial drugs (an artemisinin-based combination) as per the national treatment guidelines.

#### Qualitative phase

Key informant interview was conducted among the leaders of head porters in all three markets with the aid of an interview guide. Information such as social amenities and services, malaria burden, and prevention as well as health-seeking behaviour of ‘Kayayeis’ were obtained from their leaders. Interviews lasted a maximum of 25 min and were tape-recorded.

### Data analysis

#### Quantitative phase

STATA Version 13.0. was used for the statistical analysis. Frequencies, proportions and means (standard deviation) were used to summarize socio-demographic variables and knowledge and awareness of malaria. The dependent variable (malaria infection) was categorized as presence or absence of malaria parasites in the blood. Associations between the dependent and independent variables were determined using a simple logistic regression, after which variables showing significance at the crude level were subsequently used in a multiple logistic regression to determine their independent effects. Type of housing was later classified into two groups, good housing, and poor housing. Poor housing included shed, kiosk, and veranda/Open space, whilst good housing, included detached and semi-detached houses. Statistical significance was set at < 0.05 for all analyses.

#### Qualitative phase

All key informant interviews were conducted in local languages and tape recorded. The recordings were later translated into English, typed in Microsoft Word, and imported into NVIVO 12 software for thematic content analysis.

### Ethical consideration

The study was granted ethical approval by the Navrongo Health Research Ethics Review Committee (NHRCIRB318). Permission was sought from community opinion leaders, leaders of head porters and leaders of the various markets. Participants were fully informed about the purpose, procedures, risks, and benefits of participating in the study. Participants who were unable to read and write had their consent form read and explained to them in the presence of a trusted relative or community member. Participants who consented to be part of the study were made to sign or thumbprint the consent form as a sign of their willingness to participate in the study. Participants who tested positive to malaria RDT were given anti-malarial medications by a nurse, in accordance with the malaria case-management guidelines of the NMEP and GHS.

## Results

### Background characteristics of head porters

In all, 754 participants were recruited in this study, with 48.54% (366/754) of the participants recruited from Accra. The median age of participants was 23yrs (Range 18–30 yrs), and most 60.88% (459/754) porters were below 26 years of age. Majority 67.11% (506/754) had no formal education and most 58.49% (44/754) were married. In terms of sleeping places, a third 37.40% (282/754) of them slept on veranda/open spaces, and a quarter 25.07% (189/754) slept in kiosks (wooden or metallic containers usually improvised as homes, presenting with challenges including limited space, poor ventilation and no mosquito screens) (Table 1). The accommodation situation looked different across the three study sites. In Accra, 71.3% slept on veranda/open space compared to 14.1% in Tamale and 16.7% in Kumasi. Fifty-four percent of participants in Kumasi, 6.7% in Tamale and 4.1% in Accra lived in kiosk. Whilst, 41.6% in Tamale, 11.7% Kumasi and 9.0% Accra lived in semi-detached buildings (Table 2). Porters in Tamale had relatively better accommodation than those in those in Accra and Kumasi (p = 0.001) (Table 3).

**Table 1:** Background characteristics of head porters

Variable	Frequency (N = 754)	%
City of operation		
Accra	366	48.54
Kumasi	239	31.7
Tamale	149	19.76
Market of operation		
Aboabo	75	9.95
Bombay	73	9.68
Central market	149	19.76
KTI	91	12.07
Malata	181	24.01
Tema station/makola	185	24.54
Age (years)		
≤ 25	459	60.9
26–35	117	23.4
≥ 36	118	15.7
Marital status		
Cohabiting	40	5.31
Divorced	8	1.06
Married	441	58.49
Separated	9	1.19
Single	256	33.95
Level of formal education		
JHS/MSLC	43	5.70
No formal education	506	67.11
Primary	170	22.55
SHS/vocational	35	4.64
Type of housing		
Detached house	64	8.48
Kiosk	189	25.07
Semi-detached house	152	20.16
Shed	67	8.89
Varanda/open space	282	37.4
Religion		
Christian	34	4.51
Muslim	717	95.09
None	3	0.40
Ever given birth		
Yes	479	63.53
No	275	36.47
Number of children		
1–2	225	46.97
3–4	155	32.36
5–6	84	17.54
≥ 7	12	2.71
None	2	0.42
Pregnancy status		
Yes	53	7.03
No	685	90.85
Not sure	16	2.12
Trimester of pregnancy (N = 53)		
First	25	47.17
Second	19	35.85
Third	8	15.09
Don’t know	1	1.89
Attended ANC (N = 53)		
Yes	30	56.6
No	23	43.4
Taken SP (N = 53)		
Yes	22	41.51
No	31	58.49

**Table 2:** Type of accommodation by city

Type of housing	Accra	Kumasi	Tamale
Detached	0 (0.0)	35 (14.6)	29 (19.5)
Semi-detached	33 (9.0)	28 (11.7)	62 (41.6)
Varanda/open space	261 (71.3)	40 (16.7)	21 (14.1)
Kiosk	15 (4.1)	130 (54.4)	10 (6.7)
Shed	53 (14.5)	1 (0.4)	13 (8.7)
Other	4 (1.1)	5 (2.1)	14 (9.4)

**Table 3:** Differences between city and type of housing among head porters, Ghana

City	Type of housing		cOR	P-value
	Good housing	Poor housing		
Accra	33 (9.1)	333 (91.9)	0.063 (0.039, 0.103)	< 0.001
Kumasi	63 (26.4)	176 (73.6)	0.228 (0.147, 0.353)	< 0.001
Tamale	91 (61.6.)	58 (38.9)	1.0	

### Malaria prevalence and health-seeking behaviour of head porters

**Fig. 1:**
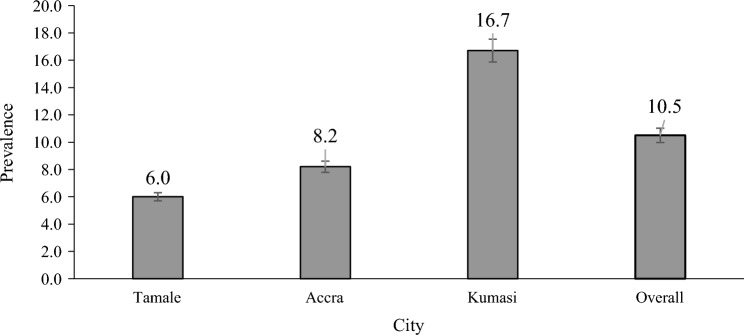
Prevalence of malaria among head porters

The overall prevalence of malaria among head porters was 10.48% (79/754) with varied prevalence levels across the three cities as follows: Kumasi recorded the highest prevalence of 16.7% (40/239), followed by Accra 8.2% (30/366) and the least been 6.0% in Tamale (Fig. 1). Nearly half 48.94% (369/754) of the sampled porters reported having experienced malaria in the past 6 months, of which 90.51% (334/369) of them sought care. Most 61.68% (206/334) of them sought care from hospitals/clinics and nearly a third 33.24% (111/334) of them also sought care from either pharmacies or Over the Counter Medicines outlets. Almost half 49.6% (374/754) of the porters had health insurance, with 98.66% (369/374) of them on the National Health Insurance Scheme (Table 4).

**Table 4:** History of malaria and health-seeking of head porters

Variable	Frequency (N = 754)	%
Had malaria in past 6 months		
Yes	369	48.94
No	385	51.06
Sought care		
Yes	334	90.51
No	35	9.49
Type of care sought		
Herbalist/herbal shops	7	2.09
Hospital/clinic	206	61.68
Mobile drug vendors	10	2.99
OTCM/pharmacy	111	33.24
Malaria confirmation		
Yes	219	65.56
No	115	34.44
Medicine taken		
Prescribed antimalarial	264	79.04
Took antimalarial without prescription	60	17.96
Took herbal medicine	8	2.40
Did not take any medicine	2	0.60
Belong to Health Insurance Scheme		
Yes	374	49.6
No	380	50.4
Type of Health Insurance (N = 374)		
Mutual Health organization/community	5	1.34
NHIS	369	98.66
Aware NHIS cover malaria treatment		
Yes	429	56.9
No	325	43.1

## The malaria burden among the porters was also exemplified below by their leaders

“The main health problem among Kayayeis is malaria because Kayayeis sleep outside on these pavements and verandas which exposes them to mosquito bites and even rain when it rains” (Leader 1, Accra).

*“Most Kayayeis permanently sleep outside which exposes them to mosquito bites. So, malaria always attacks Kayayeis (Leader 3, Accra)”.*

### Malaria knowledge and use of long-lasting insecticidal nets (LLIN) among head porters

Most 84.48% (637/754) head porters have ever heard of malaria, and more than half 58.4% (372/637) of them had received malaria education in the past 6 months. More than a third 40.6% (259/637) of those aware of malaria knew how to protect themselves from malaria infections. Regarding LLIN, 27.5% (207/754) of head porters owned at least one net, and majority 77.78% (161/207) of those with nets slept under them a night before the survey (Table 5).

**Table 5:** Malaria knowledge, LLIN ownership and use among head porters in Ghana

Variable	Frequency	%
Ever heard about malaria		
Yes	637	84.48
No	117	15.52
Had malaria education in past 6 months		
Yes	372	58.4
No	265	41.6
Know how to protect		
Yes	259	34.4
No	378	65.6
Have LLIN		
Yes	207	27.45
No	547	72.55
Source of LLIN		
Bought it from a shop	1	0.48
Gift from family	2	0.97
Health worker through mass distribution	120	57.97
Health workers at ANC	29	14.01
Health workers at CWC	16	7.73
NGOs	19	9.18
Other	14	6.76
Schools	6	2.9
When LLIN was received		
1–2 years	32	15.46
3 years	5	2.42
Above 3 years	5	2.42
Less than 1 year	165	79.71
Slept under LLIN last night		
Yes	161	77.78
No	46	22.22

### Factors associated with malaria parasitaemia

Receiving malaria education in the past 6 months and having a good knowledge of malaria were factors associated with testing positive for malaria in this study. Participants who received education on malaria within 6 months preceding the survey had 52% reduced odds of testing positive for malaria than those who had not been educated [AOR = 0.48 (0.26–0.88), p 0.02]. Also, those who had poor malaria knowledge had 2.03 higher odds of testing positive to malaria than those who had good malaria knowledge [2.23 (1.26–4.27), p < 0.01] (Table 6).

**Table 6:** Logistic regression analysis of factors associated with malaria among head porters

Variable	Malaria status		Unadjusted odd ratio		Adjusted odd ratio	
	Positive	Negative	OR (95% CI)	P value	OR (95% CI)	P value
Family slept under ITN						
Yes	11	525	0.29 (0.11–0.74)	0.002	0.72 (0.39–1.35)	0.30
No	11	150				
Had malaria in past 6 months						
Yes	25	360	0.41 (0.24–0.68)	< 0.001	1.14 (0.64–2.04)	0.658
No	54	315				
Had malaria education in past 6 months						
Yes	20	245	0.58 (0.32–1.03)	0.049	0.48 (0.26–0.88)	0.02
No	46	326				
Involved in past educational message						
Yes	53	584	0.32 (0.18–0.56)	< 0.01	0.80 (0.37–1.73)	0.576
No	26	91				
Marital status						
Married	38	235	1.74 (1.05–2.85)	0.020	1.75 (0.997–3.10)	0.05
Single	41	440				
Malaria knowledge						
Poor	25	131	2.03 (1.14–3.56)	< 0.01	2.23 (1.26–4.27)	< 0.01
Good	41	436				

Knowledge of malaria among the leaders of the porters is stated below:

#### Cause

“I have heard that dirt breeds mosquitoes which gives malaria. You can see that the gutters in this community are filled with filth and refuse all over. Always, there are lots of mosquitoes here. Therefore, malaria cases are a lot” (Leader 1, Kumasi).

“Mosquitoes and dirty places like the dirty gutter causes malaria. The rain falls in these gutters… they all cause malaria” (Leader 2, Accra).

“The dirty environment is the first and major cause of malaria. Secondly, if you don’t have mosquito net or the spray or coil, you easily get malaria” (Leader 1, Accra).

#### Symptoms

“The symptoms I know are headache and vomiting (Leader 1, Accra).

Headaches and body pains but if one doesn’t treat it early it will make him or her very weak and as a result won’t be able to go to work” (Leader 2, Accra).

#### Prevention of malaria

Leaders of the head porters indicated ways malaria could be prevented as they shared with us:

“Firstly, malaria is prevented through general cleaning of the market. Again, using mosquito nets distributed by health workers” (Leader 2, Accra).

“Sleeping under mosquito nets. Some of the Kayayeis with mosquito nets sleep in it to protect themselves from malaria, others also use mosquito coils to prevent malaria” (Leader 1, Kumasi).

## Discussion

Mobile populations present a significant challenge for public interventions, posing a substantial danger to the elimination efforts of many transmissible illnesses, including malaria. They serve as reservoirs that drive transmission of malaria, particularly in areas where cases are low. This study sought to determine prevalence and factors associated with malaria among head porters in Ghana to inform decision-making. Malaria prevalence among the porters was 10.5%, with varied prevalence across the three cities. Kumasi recorded the highest prevalence of 16.7% followed by Accra 8.2% and 6.0% in Tamale. The overall malaria prevalence found in this study was lower than the 14.1% national estimate reported by the 2019 Malaria Indicator Survey (MIS) carried out in same year. The relatively low prevalence could have resulted from the different age groups involved in the two studies. Participants of the MIS were children less than six years whilst median age of participants in this study was 23yrs. Children particularly, those under the age of five years have higher risk of malaria due to their fragile immunity and that may have led to the relatively lower prevalence in this study than the MIS. The prevalence in this study is also lower compared to that found by Kwofie et al*.* [13], which reported 12% prevalence of malaria infection among head porters. Further, a study by Diallo et al*.* [14] found 15.1% prevalence of malaria among hawkers and long-distance truck drivers in the Greater Accra Region of Ghana, which is comparatively higher than the prevalence of this study. This low parasitaemia might have resulted from the difference in the period of sample collection between the two studies. As samples for this study was collected in November (Minor malaria season) whilst that of Diallo et al*.* [14] was done June to July (Major malaria season). The parasitaemia level was also lower than that 35%, as reported by Erhabor et al. [15] in Nigeria, and 18.4% as reported by Abebaw et al*.* [16] in Ethiopia.

The study found a lower prevalence in Tamale than that of Accra and Kumasi, which may have resulted from the accommodation differences between Tamale porters and that of the two cities. The data suggests that porters in Tamale had a relatively good accommodation compared to those in Accra and Kumasi. And this might have led to the comparatively higher prevalence in Tamale than the other two cities, highlighting the role of housing play in malaria transmission [17]. Further, the 8.0% prevalence found in Accra is about 3.3 times the regional average (2.4%) reported by the MIS in 2019. This reflects their increased risk to malaria due to lack of proper accommodation facilities for this population in the business city of Accra.

Receiving malaria education 6 months prior to the study and having a good knowledge of malaria were protective factors against malaria in this study. The increased knowledge in malaria may have empowered them to act right, by using available protective measures to protect themselves from malaria. They may also seek appropriate treatment for themselves and their dependants when they have malaria. This finding agrees with a study carried out in Ethiopia by Tesfahunegn et al. which indicated participants who had a good knowledge of malaria had a reduced odds of testing positive for malaria than those who had poor knowledge about the disease [18]. Further, a similar study also in Ethiopia found insufficient knowledge in malaria as a predictor of malaria among the study population [19]. Most participants were below 40 years and not registered with any health insurance scheme. This could make health seeking very difficult due to their poor economic status. This aligns with a recent study done among same population in Accra as findings indicate a significant proportion of head porters were younger than 40 years and are unlikely to experience the protective advantages associated with older age and healthcare. This observation raises concerns regarding the effectiveness of intervention strategies aimed at addressing the needs of this specific population of economic migrants [13]. Opuni et al*.* [10] assert that specific health needs should be included in any health intervention targeting head porters.

This study had a few limitations. Firstly, Rapid Diagnostic Testing (RDT) method was used for the diagnosis of malaria hence participants who have been treated for malaria within the last few weeks who are not necessarily carrying the malaria parasites at the time of testing may still test positive for malaria. Secondly, there is the possibility of recall/information bias since some participants are more or less likely to recall and relate information on some exposures. There is also a possibility of misinformation/misclassification of accommodation of some participants as this was not verified by the collectors.

## Conclusion

This study reports malaria prevalence of 10.5% among head porters in Ghana. Majority of the porters were not registered with any health insurance scheme, and most slept outside on pavements and verandas. Receiving malaria education in the past 6 months reduced the odds of malaria infection among the head porters whiles poor knowledge of malaria increased the odds of malaria infection among them. The National Malaria Elimination Programme and partners should provide Long-lasting insecticidal nets (LLIN) and other outdoor interventions for use by this special group. Designated state institutions should arrange free National Health Insurance Scheme (NHIS) registration for ‘Kayayeis’ to narrow the health access gap.

## Data Availability

Data supporting the conclusions made have been included in the article. The dataset for this study will be made available upon reasonable request to the corresponding author.

## Abbreviations

ACT: Artemisinin-based combination therapy
ANC: Antenatal clinic
CWC: Child welfare clinic
DE: Design effect
FY: Fiscal year
GDHS: Ghana Demographic and Health Survey
GHS: Ghana Health Service
GSS: Ghana Statistical Service
HPM: Human population movement
HRP2: Histidine-rich protein 2
KTI: Kumasi Technical Institute
LLIN: Long lasting insecticidal net
MICS: Multiple Indicator Cluster Survey
MIS: Malaria Indicator Survey
NHIS: National Health Insurance Scheme
NHRCIRB: Navrongo Health Research Centre Institutional Review Board
NGO: Non-Governmental Organization
ODK: Open data kit
JHS: Junior High School
RDT: Rapid diagnostic test
RDS: Respondent driven sampling
SHS: Senior High School
WHO: World Health Organization
